# Disparities in Adverse Maternal Outcomes Among Asian Women in the US Delivering at Term

**DOI:** 10.1001/jamanetworkopen.2020.20180

**Published:** 2020-10-09

**Authors:** Stephen M. Wagner, Matthew J. Bicocca, Megha Gupta, Suneet P. Chauhan, Hector Mendez-Figueroa, Jacqueline G. Parchem

**Affiliations:** 1 Department of Obstetrics, Gynecology, and Reproductive Sciences, McGovern Medical School, University of Texas Health Science Center at Houston

## Abstract

This population-based retrospective cohort study used US Vital Statistics data from 2014-2017 to assess the risk of adverse maternal outcomes at term for Asian women in the US.

## Introduction

Disparities in maternal outcomes are representative of racial/ethnic health inequities in the US.^1^ Recent data suggest that Asian women, even those considered at low risk, have higher rates of certain adverse outcomes in the peripartum period compared with White women.^2^ Many studies are limited regarding outcomes for Asian women, either excluding them entirely or grouping diverse ethnicities despite known differences in culture, lifestyle, comorbidities, and risk.^3,4,5^ Given the understudied nature of this increasing demographic of the US population, our objective was to assess the risk of maternal adverse outcomes at term for Asian women in the US according to ethnicity.

## Methods

This population-based retrospective cohort study used US Vital Statistics data from 2014-2017. The study population was restricted to women with nonanomalous singleton pregnancies who labored and had live births between 37 and 41 completed weeks of gestation. Women whose self-reported race was Asian or White according to the 2003 revised birth certificate were included. Asian women were further subdivided by self-reported ethnicity: Asian Indian, Chinese, Filipina, Japanese, Korean, Pacific Islander, Vietnamese, or other. Institutional review board approval for the study was obtained from the University of Texas Health Science Center at Houston with a waiver of informed consent.

The main exposure variable was maternal race/ethnicity. Non-Hispanic White women served as the reference group. The primary outcome was a composite of adverse maternal outcomes, defined as any of the following: obstetric anal sphincter injury, admission to the intensive care unit, maternal blood transfusion, uterine rupture, or unplanned hysterectomy. Because of the high prevalence of obstetric anal sphincter injury relative to the other components of the composite outcome, a sensitivity analysis was performed with this injury excluded.

Maternal characteristics were compared using the χ^2^ test for categorical variables. Rates of the composite outcome were reported as the number of cases per 1000 live births. Multivariable Poisson regression models were used to estimate the association between racial/ethnic group and the composite outcome while adjusting for possible confounders (eMethods in the Supplement).

## Results

For 15.8 million live births, 8 815 877 (56.5%) women met inclusion criteria. Of these women, 758 709 (8.6%) were Asian and 8 057 168 (91.4%) were White. Significant differences in baseline characteristics were observed between groups, including differences in education, marital status, insurance, prenatal care, and smoking (Table 1). Vaginal delivery rates were similar for Pacific Islander and Japanese women compared with White women, but lower for other groups. Women of all Asian ethnic groups had higher rates of gestational diabetes, but generally lower rates of neonates large for gestational age.

**Table 1.** Baseline Characteristics

	No. (%)										*P* value
	Total (n = 8 815 877)	Maternal ethnicity									
		Asian Indian (n = 196 640)	Chinese (n = 177 949)	Filipino (n = 86 825)	Japanese (n = 21 192)	Korean (n = 45 203)	Other Asian (n = 139 201)	Pacific Islander (n = 32 033)	Vietnamese (n = 59 666)	White (n = 8 057 168)	
Maternal age, y											<.001
<20	510 194 (5.8)	1482 (0.8)	882 (0.5)	1529 (1.8)	109 (0.5)	308 (0.7)	4632 (3.3)	2311 (7.2)	781 (1.3)	498 160 (6.2)	
≥35	1 317 282 (14.9)	31 946 (16.2)	46 894 (26.4)	23 936 (27.6)	9481 (44.7)	15 556 (34.4)	23 769 (17.1)	3912 (12.2)	14 738 (24.7)	1 147 050 (14.2)	
Maternal education											<.001
<High school	1 186 513 (13.5)	10 530 (5.4)	12 661 (7.1)	3362 (3.9)	525 (2.5)	942 (2.1)	26 573 (19.1)	8053 (25.1)	6113 (10.2)	1 117 754 (13.9)	
≥High school	7 526 967 (85.4)	182 159 (92.6)	160 015 (89.9)	81 043 (93.3)	20 169 (95.2)	43 416 (96.0)	109 271 (78.5)	23 177 (72.4)	51 592 (86.5)	6 856 125 (85.1)	
Unknown	102 397 (1.2)	3951 (2)	5273 (3)	2420 (2.8)	498 (2.3)	845 (1.9)	3357 (2.4)	803 (2.5)	1961 (3.3)	83 289 (1.0)	
Marital status											<.001
Married	5 622 604 (63.8)	177 737 (90.4)	139 512 (78.4)	60 138 (69.3)	17 995 (84.9)	38 344 (84.8)	96 130 (69.1)	15 486 (48.3)	43 062 (72.2)	5 034 200 (62.5)	
Unknown	294 879 (3.3)	9667 (4.9)	19 302 (10.8)	8145 (9.4)	1431 (6.8)	3111 (6.9)	7531 (5.4)	1375 (4.3)	4703 (7.9)	239 614 (3.0)	
Nulliparous	3 179 026 (36.1)	91 088 (46.3)	78 153 (43.9)	33 886 (39.0)	8528 (40.2)	19 738 (43.7)	48 316 (34.7)	9180 (28.7)	24 294 (40.7)	2 865 843 (35.6)	<.001
Insurance											<.001
Government	3 500 508 (39.7)	39 466 (20.1)	45 555 (25.6)	22 828 (26.3)	3036 (14.3)	9002 (19.9)	67 796 (48.7)	19 174 (59.9)	21 927 (36.7)	3 271 724 (40.6)	
Private	4 673 177 (53.0)	148 695 (75.6)	98 229 (55.2)	58 927 (67.9)	16 865 (79.6)	33 516 (74.1)	63 891 (45.9)	8573 (26.8)	34 588 (58.0)	4 209 893 (52.3)	
Other/unknown	642 192 (7.3)	8479 (4.3)	34 165 (19.2)	5070 (5.8)	1291 (6.1)	2685 (5.9)	7514 (5.4)	4286 (13.4)	3151 (5.3)	575 551 (7.1)	
Prenatal care											<.001
Yes	8 518 118 (96.6)	190 173 (96.7)	173 729 (97.6)	84 349 (97.1)	20 535 (96.9)	43 757 (96.8)	133 913 (96.2)	29 628 (92.5)	57 594 (96.5)	7 784 440 (96.6)	
Unknown	200 059 (2.3)	5153 (2.6)	3463 (1.9)	1875 (2.2)	537 (2.5)	1234 (2.7)	3771 (2.7)	1310 (4.1)	1429 (2.4)	181 287 (2.3)	
Smoking during pregnancy											<.001
Yes	634 861 (7.2)	317 (0.2)	396 (0.2)	931 (1.1)	188 (0.9)	553 (1.2)	1850 (1.3)	1359 (4.2)	317 (0.5)	628 950 (7.8)	
Unknown	54 947 (0.6)	583 (0.3)	642 (0.4)	1804 (2.1)	767 (3.6)	352 (0.8)	994 (0.7)	1310 (4.1)	285 (0.5)	48 210 (0.6)	
Prepregnancy BMI category^a^											<.001
Underweight	334 873 (3.8)	10 013 (5.1)	23 273 (13.1)	4650 (5.4)	2316 (10.9)	3911 (8.7)	7884 (5.7)	633 (2)	7244 (12.1)	274 949 (3.4)	
Normal weight	4 247 736 (48.2)	110 585 (56.2)	121 956 (68.5)	49 743 (57.3)	14 802 (69.8)	31 371 (69.4)	74 570 (53.6)	9155 (28.6)	41 150 (69)	3 794 404 (47.1)	
Overweight	2 193 063 (24.9)	52 646 (26.8)	19 752 (11.1)	20 644 (23.8)	2487 (11.7)	6563 (14.5)	34 335 (24.7)	8640 (27.0)	7303 (12.2)	2 040 693 (25.3)	
Class I obese	1 071 707 (12.2)	14 282 (7.3)	3958 (2.2)	7092 (8.2)	714 (3.4)	1671 (3.7)	12 455 (8.9)	6413 (20.0)	1817 (3)	1 023 305 (12.7)	
Class II obese	471 637 (5.3)	2973 (1.5)	813 (0.5)	1874 (2.2)	192 (0.9)	401 (0.9)	3487 (2.5)	3475 (10.9)	417 (0.7)	458 005 (5.7)	
Class III obese	279 062 (3.2)	810 (0.4)	255 (0.1)	671 (0.8)	73 (0.3)	104 (0.2)	1133 (0.8)	2153 (6.7)	175 (0.3)	273 688 (3.4)	
Unknown	217 799 (2.5)	5331 (2.7)	7942 (4.5)	2151 (2.5)	608 (2.9)	1182 (2.6)	5337 (3.8)	1564 (4.9)	1560 (2.6)	192 124 (2.4)	
Prior cesarean											<.001
Yes	300 009 (3.4)	9605 (4.9)	7103 (4)	3036 (3.5)	596 (2.8)	1263 (2.8)	5989 (4.3)	1791 (5.6)	1880 (3.2)	268 746 (3.3)	
Unknown	864 (0.0)	8 (0.0)	8 (0.0)	15 (0.0)	2 (0.0)	2 (0.0)	22 (0.0)	10 (0.0)	3 (0.0)	794 (0.0)	
Delivery method											<.001
Vaginal	7 441 638 (84.4)	145 168 (73.8)	145 179 (81.6)	69 275 (79.8)	17 941 (84.7)	36 853 (81.5)	115 393 (82.9)	27 156 (84.8)	48 625 (81.5)	6 836 048 (84.8)	
Forceps	65 067 (0.7)	2357 (1.2)	1475 (0.8)	796 (0.9)	303 (1.4)	458 (1.0)	1191 (0.9)	278 (0.9)	443 (0.7)	57 766 (0.7)	
Vacuum	311 670 (3.5)	13 457 (6.8)	11 297 (6.3)	4389 (5.1)	1078 (5.1)	2570 (5.7)	6326 (4.5)	961 (3)	3707 (6.2)	267 885 (3.3)	
Cesarean	996 816 (11.3)	35 643 (18.1)	19 987 (11.2)	12 361 (14.2)	1869 (8.8)	5321 (11.8)	16 284 (11.7)	3637 (11.4)	6889 (11.5)	894 825 (11.1)	
Unknown	686 (0.0)	15 (0.0)	11 (0.0)	4 (0.0)	1 (0.0)	1 (0.0)	7 (0.0)	1 (0.0)	2 (0.0)	644 (0.0)	
Pregestational diabetes	42 849 (0.5)	1572 (0.8)	689 (0.4)	680 (0.8)	70 (0.3)	223 (0.5)	912 (0.7)	302 (0.9)	412 (0.7)	37 989 (0.5)	<.001
Gestational diabetes	464 518 (5.3)	23 042 (11.7)	16 101 (9.0)	8825 (10.2)	1236 (5.8)	3204 (7.1)	13 047 (9.4)	2240 (7)	6123 (10.3)	390 700 (4.9)	<.001
Chronic hypertension	92 809 (1.1)	1179 (0.6)	610 (0.3)	1290 (1.5)	124 (0.6)	300 (0.7)	933 (0.7)	343 (1.1)	252 (0.4)	87 778 (1.1)	<.001
Gestational hypertension or preeclampsia	418 828 (4.8)	5695 (2.9)	2967 (1.7)	4278 (4.9)	489 (2.3)	1107 (2.4)	4010 (2.9)	1465 (4.6)	1276 (2.1)	397 541 (4.9)	<.001
Eclampsia	12 343 (0.1)	153 (0.1)	102 (0.1)	242 (0.3)	37 (0.2)	46 (0.1)	139 (0.1)	133 (0.4)	45 (0.1)	11 446 (0.1)	<.001
Gestational age, wk											<.001
37	741 552 (8.4)	17 631 (9.0)	12 545 (7.0)	10 255 (11.8)	1646 (7.8)	3054 (6.8)	13 043 (9.4)	3026 (9.4)	5941 (10.0)	674 411 (8.4)	
38	1 530 175 (17.4)	39 565 (20.1)	34 298 (19.3)	21 122 (24.3)	4171 (19.7)	7875 (17.4)	28 458 (20.4)	6538 (20.4)	14 393 (24.1)	1 373 755 (17.1)	
39	3 359 092 (38.1)	73 784 (37.5)	70 687 (39.7)	32 787 (37.8)	8099 (38.2)	17 276 (38.2)	51 552 (37)	11 520 (36)	23 550 (39.5)	3 069 837 (38.1)	
40	2 418 148 (27.4)	51 302 (26.1)	48 922 (27.5)	18 388 (21.2)	5770 (27.2)	13 256 (29.3)	35 511 (25.5)	8249 (25.8)	13 003 (21.8)	2 223 747 (27.6)	
41	766 910 (8.7)	14 358 (7.3)	11 497 (6.5)	4273 (4.9)	1506 (7.1)	3742 (8.3)	10 637 (7.6)	2700 (8.4)	2779 (4.7)	715 418 (8.9)	
Birth weight											<.001
SGA	802 188 (9.1)	36 756 (18.7)	19 627 (11.0)	11 400 (13.1)	3336 (15.7)	4791 (10.6)	20 247 (14.5)	3168 (9.9)	8336 (14.0)	694 527 (8.6)	
AGA	7 177 875 (81.4)	152 230 (77.4)	149 347 (83.9)	70 636 (81.4)	17 043 (80.4)	37 618 (83.2)	111 899 (80.4)	25 400 (79.3)	48 957 (82.1)	6 564 745 (81.5)	
LGA	835 814 (9.5)	7654 (3.9)	8975 (5.0)	4789 (5.5)	813 (3.8)	2794 (6.2)	7055 (5.1)	3465 (10.8)	2373 (4.0)	797 896 (9.9)	
Year											<.001
2014	2 153 498 (24.4)	40 662 (20.7)	42 245 (23.7)	20 963 (24.1)	5126 (24.2)	10 655 (23.6)	31 774 (22.8)	7761 (24.2)	13 924 (23.3)	1 980 388 (24.6)	
2015	2 215 562 (25.1)	46 379 (23.6)	40 137 (22.6)	21 736 (25.0)	5458 (25.8)	11 153 (24.7)	33 304 (23.9)	8170 (25.5)	14 923 (25.0)	2 034 302 (25.2)	
2016	2 249 361 (25.5)	54 048 (27.5)	48 980 (27.5)	22 160 (25.5)	5414 (25.5)	12 026 (26.6)	37 034 (26.6)	7962 (24.9)	15 285 (25.6)	2 046 452 (25.4)	
2017	2 197 456 (24.9)	55 551 (28.3)	46 587 (26.2)	21 966 (25.3)	5194 (24.5)	11 369 (25.2)	37 089 (26.6)	8140 (25.4)	15 534 (26.0)	1 996 026 (24.8)	

Abbreviations: AGA, appropriate for gestational age; BMI, body mass index (calculated as weight in kilograms divided by height in meters squared); LGA, large for gestational age; SGA, small for gestational age.

^a^ Underweight, ≤18.5; normal weight, 18.5-24.9; overweight, 25.0-29.9; class I obese, 30.0-34.9; class II obese, 35.0-39.9; class III obese, ≥40.0.

The risk for maternal morbidity among Asian women varied by ethnic group (Table 2). In the adjusted model, the risk of the composite adverse outcome was higher for all groups, except for Japanese women, compared with White women. Asian Indian mothers were most likely to experience the composite maternal outcome; the predominant component was obstetric anal sphincter injury (28.7 per 1000 live births). In the sensitivity analysis, increased risk persisted only for Filipina and Pacific Islander women.

**Table 2.** Composite Maternal Morbidity by Ethnicity^a^

Outcome	Maternal ethnicity	Total live births	No.	Rate/1000 live births	RR (95% CI)	
					Unadjusted	Adjusted
Composite maternal outcome^b^	Total	8 815 877	128 052	14.5 (14.4-14.6)		
	White	8 057 168	110 588	13.7 (13.6-13.8)	1 [Reference]	1 [Reference]
	Asian Indian	196 640	6128	31.2 (30.4-31.9)	2.27 (2.21-2.33)	1.71 (1.66-1.75)
	Chinese	177 949	3871	21.8 (21.1-22.4)	1.58 (1.54-1.64)	1.25 (1.21-1.29)
	Other Asian	139 201	2841	20.4 (19.7-21.2)	1.49 (1.43-1.54)	1.44 (1.39-1.50)
	Filipina	86 825	1690	19.5 (18.6-20.4)	1.42 (1.35-1.49)	1.25 (1.20-1.31)
	Vietnamese	59 666	1249	20.9 (19.8-22.1)	1.53 (1.44-1.61)	1.28 (1.21-1.36)
	Korean	45 203	880	19.5 (18.2-20.8)	1.42 (1.33-1.51)	1.10 (1.03-1.17)
	Pacific Islander	32 033	426	13.3 (12.1-14.6)	0.97 (0.88-1.06)	1.18 (1.08-1.30)
	Japanese	21 192	379	17.9 (16.1-19.8)	1.30 (1.18-1.44)	1.09 (0.99-1.21)
Composite maternal outcome excluding OASIS^c^	Total	8 815 877	25 499	2.9 (2.9-2.9)		
	White	8 057 168	23 118	2.9 (2.8-2.9)	1 [Reference]	1 [Reference]
	Asian Indian	196 640	525	2.7 (2.4-2.9)	0.93 (0.85-1.01)	0.97 (0.89-1.06)
	Chinese	177 949	456	2.6 (2.3-2.8)	0.89 (0.81-0.98)	0.95 (0.86-1.04)
	Other Asian	139 201	565	4.1 (3.7-4.4)	1.41 (1.30-1.54)	1.44 (1.32-1.56)
	Filipina	86 825	329	3.8 (3.4-4.2)	1.32 (1.18-1.47)	1.35 (1.21-1.51)
	Vietnamese	59 666	173	2.9 (2.5-3.4)	1.01 (0.87-1.17)	1.05 (0.90-1.22)
	Korean	45 203	136	3.0 (2.5-3.6)	1.05 (0.89-1.24)	1.12 (0.95-1.33)
	Pacific Islander	32 033	143	4.5 (3.8-5.3)	1.56 (1.32-1.83)	1.46 (1.24-1.72)
	Japanese	21 192	54	2.5 (1.9-3.3)	0.89 (0.68-1.16)	0.95 (0.73-1.24)

Abbreviations: OASIS, obstetric anal sphincter injury; RR, relative risk.

^a^ Composite maternal outcome includes admission to intensive care unit, blood transfusion, uterine rupture, unplanned hysterectomy, or third- or fourth-degree laceration unless otherwise specified.

^b^ Adjusted for maternal age, education, marital status, insurance type, nulliparity, prior cesarean delivery, body mass index, prenatal care, smoking, newborn weight, operative delivery, diabetes, hypertension, eclampsia, and birth year.

^c^ Adjusted for maternal age, education, marital status, insurance type, nulliparity, prior cesarean delivery, body mass index, prenatal care, smoking, diabetes, hypertension, eclampsia, and birth year.

## Discussion

Our results indicate variability in maternal risk across Asian ethnic groups, with an increased risk for most groups compared with White women. This increased risk was strongly driven by obstetric anal sphincter injury, which has previously been associated with Asian race/ethnicity.^6^ The reasons that Filipina and Pacific Islander women remained at increased risk in the sensitivity analysis are unknown and deserve further attention. In particular, research on social determinants of health, access to care, and structural racism is needed.^1^ This analysis was limited to maternal outcomes reported in the data set; thus, some outcomes could not be analyzed. Our study highlights the need for specific data on racial/ethnic subgroups to fully appreciate maternal health disparities.

## Notes

Supplement. **eMethods.**
