# Supplementary material for: Disparities in Adverse Maternal Outcomes Among Asian Women in the US Delivering at Term
Source: JAMA Netw Open. 2020 Oct 9;3(10):e2020180. doi: 10.1001/jamanetworkopen.2020.20180 (PMC7547364; doi:10.1001/jamanetworkopen.2020.20180)
Supplement: Supplement. — eMethods. [file jamanetwopen-e2020180-s001.pdf]

Supplemental Online Content

Wagner SM, Bicocca MJ, Gupta M, Chauhan SP, Mendez-Figueroa H, Parchem JG. Disparities in adverse maternal outcomes among Asian women in the US delivering at term. *JAMA Netw Open*. 2020;3(10):e2020180. doi:10.1001/jamanetworkopen.2020.20180

eMethods.

This supplemental material has been provided by the authors to give readers additional information about their work.

© 2020 Wagner et al. *JAMA Network Open.*

Online Only Supplemental Material

eMethods.

We used Period Linked Birth-Infant Death Data of National Vital Statistics data, which are collected by the National Center for Health Statistics and published by the Center for Disease Control and Prevention (available from: https://www.cdc.gov/nchs/data_access/VitalStatsOnline.htm). Women who did not labor and whose maternal race/ethnicity was black, Hispanic, American Indian, other or unknown were excluded from the analysis.

The primary outcome was a composite maternal adverse outcome, defined as any of the following: OASIS, admission to the intensive care unit, maternal blood transfusion, uterine rupture, or unplanned hysterectomy. Women with more than one adverse outcome were counted only once when formulating the composite. Multivariable Poisson regression models with robust error variance were used to estimate the association between racial/ethnic group and the composite adverse outcome, while adjusting for possible confounders: maternal age (<20, 20-34, ≥35 years), maternal education (less than high school, high school, more than high school, unknown), marital status (married, not married), insurance type (none, government, private), nulliparous (yes, no, unknown), prior cesarean (yes, no, unknown), prenatal care (yes, no, unknown), smoking during pregnancy (yes, no, unknown), pre-pregnancy body mass index [BMI] (underweight, BMI <18.5 kg/m2; normal weight, BMI 18.5 to 24.9 kg/m^2; overweight, BMI 25.0 to 29.9 kg/m^2; obesity class I, BMI 30.0 to 34.9 kg/m^2; obesity class II, BMI of 35.0 to 39.9 kg/m^2; obesity class III, BMI of at least 40.0 kg/m^2; unknown), diabetes (yes, no, unknown),

© 2020 Wagner et al. *JAMA Network Open.*

hypertension (yes, no, unknown), eclampsia (yes, no, unknown), and birth year (2014, 2015, 2016, 2017). The results were presented as adjusted relative risk (aRR) with a 95% confidence interval (CI). All tests were two-tailed, and a p-value of <.05 was considered significant. Statistical analyses were conducted using STATA 16.

© 2020 Wagner et al. *JAMA Network Open.*
